# Catastrophic Type A Aortic Dissection Temporally Associated With Recreational Ketamine Use

**DOI:** 10.7759/cureus.97346

**Published:** 2025-11-20

**Authors:** Cobi T Bissell, Kyle Rankin

**Affiliations:** 1 Emergency Medicine, University Medical Center of Southern Nevada, Las Vegas, USA

**Keywords:** adverse effects of ketamine, aortic dissection, cardiovascular complications, emergency medicine imaging, substance recreational use, type a aortic dissection

## Abstract

Aortic dissection is a life-threatening vascular emergency, typically associated with hypertension, connective tissue disease, or stimulant drug use. Ketamine, a dissociative anesthetic with sympathomimetic properties, is rarely implicated in major cardiovascular events. This case report describes a 57-year-old woman with no known medical history who presented with acute altered mental status after reported intranasal ketamine use and subsequent back pain. On arrival, she was obtunded and hypertensive, requiring intubation for airway protection. CT angiography revealed a Type A aortic dissection extending from the root to the left iliac artery with involvement of the carotid, mesenteric, and renal vessels. Despite initial conservative management and later surgical intervention, the patient suffered intraoperative cardiac arrest and was pronounced deceased. This case highlights the potential for ketamine-induced sympathomimetic effects to precipitate vascular catastrophe in susceptible individuals.

## Introduction

Aortic dissection results from an intimal tear causing separation of the aortic wall layers, leading to catastrophic outcomes if not promptly diagnosed. Reported risk factors include hypertension, congenital aortic disease, and connective tissue disorders. Illicit stimulants such as cocaine and amphetamines are well-established precipitants due to catecholamine surges and elevated shear stress. The estimated annual incidence of aortic dissection is approximately 3 cases per 100,000 person-years, with mortality rates approaching 20-30% within the first 24 hours if untreated [1,2]. Patients typically present to the emergency department with sudden, severe chest or back pain, pulse deficits, or neurologic deficits.

Ketamine is a dissociative anesthetic of the phencyclidine derivative class that functions primarily as an NMDA receptor antagonist. It produces analgesia, amnesia, and sedation while exhibiting sympathomimetic effects through catecholamine release and reuptake inhibition, transiently increasing heart rate and blood pressure [3]. Despite its extensive medical and recreational use, its potential contribution to major vascular pathology is underrecognized. Ketamine is used medically in anesthesia, procedural sedation, and pain management, and increasingly in outpatient infusion centres for depression [4]. Recreationally, it is commonly insufflated among young adults, with chronic use associated with urologic and hepatic complications such as hemorrhagic cystitis and cholestatic injury [5]. We present a case of extensive Stanford Type A dissection temporally associated with reported ketamine use.

## Case presentation

A 57-year-old female with no reported medical history was found unresponsive at home approximately 45 minutes before emergency department (ED) arrival. Her husband briefly presented to the ED, reporting that she had insufflated ketamine approximately 10-15 minutes before the onset of severe back pain and subsequent collapse, noting a history of recurrent recreational use.

On arrival to the ED, she was obtunded with shallow respirations and a Glasgow Coma Scale (GCS) score of 3 (E1V1M1). No external trauma, abnormal movements, nystagmus, posturing, or rigidity were observed. Peripheral pulses were symmetric without deficit. Pupils were equal and reactive, and she exhibited no focal neurologic findings. Initial vital signs showed significant hypertension with a systolic blood pressure in the 180s, a heart rate of 60 bpm, and an oxygen saturation of 100% while on manual ventilation. Rapid sequence intubation was performed using etomidate and rocuronium for airway protection, given a GCS of 3 and in anticipation of further clinical deterioration.

Initial laboratory studies were notable for elevated lactate (8.7 mmol/L, reference range 0.5-2.2 mmol/L), mild normocytic anemia (hemoglobin 9.0 g/dL, reference range 12 to 16 g/dL, hematocrit 28.5%, reference range 36% to 44%), and hyperglycemia (glucose 296 mg/dL, reference range 70-99 mg/dL). Electrolytes and renal function were within normal limits, with a mild metabolic acidosis (CO₂ 21, anion gap 15). Coagulation profile, creatine kinase, BNP, and urinalysis were normal. Electrocardiogram demonstrated sinus rhythm with mild left atrial enlargement but no ischemic changes, and troponin was within normal limits. Toxicology screening was negative for cocaine and amphetamines, with benzodiazepines and fentanyl attributed to iatrogenic administration. The ethanol and respiratory BioFire panels were also negative.

**Figure 1:**
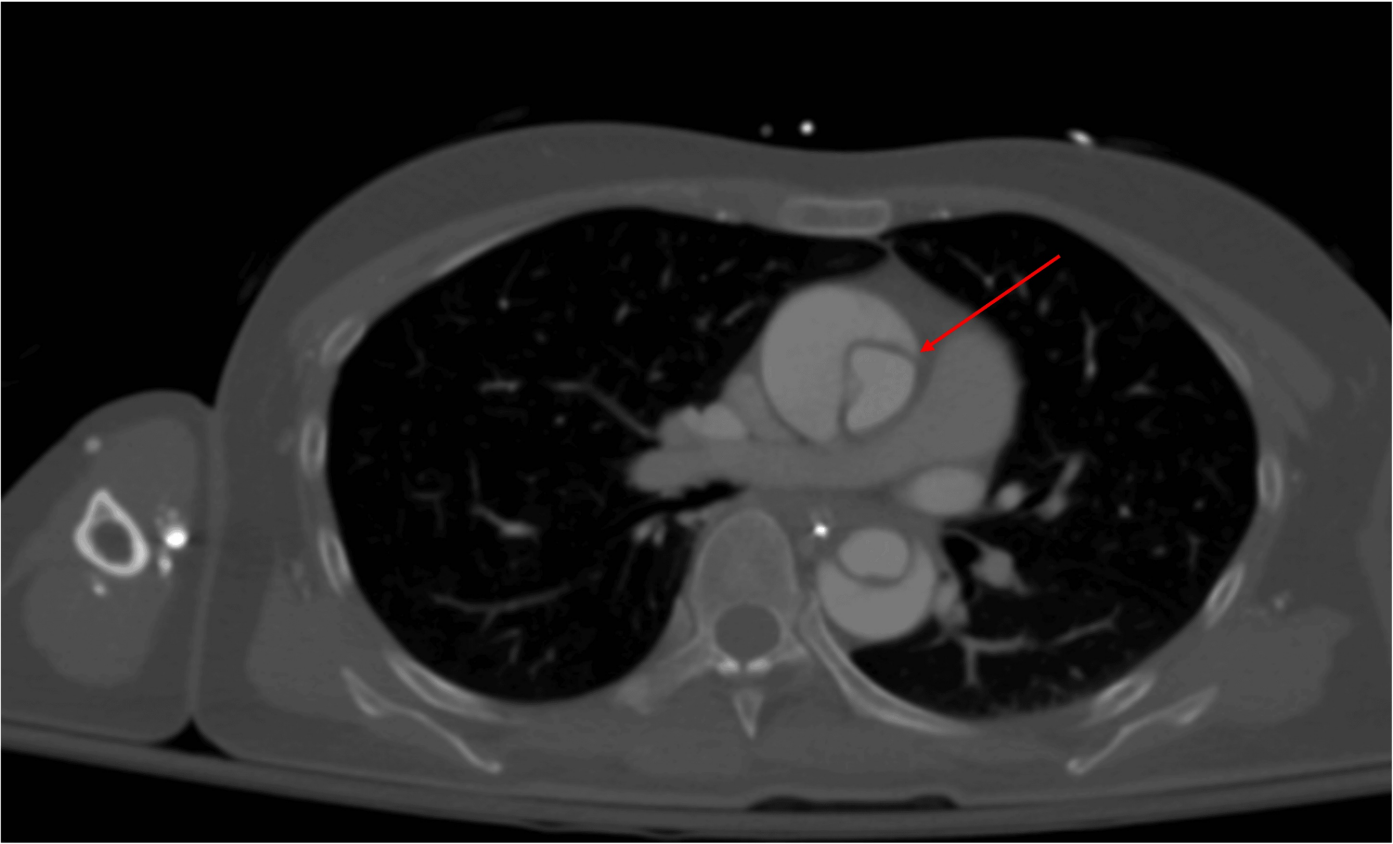
Axial CT angiogram of the chest demonstrating a Stanford Type A aortic dissection with visible intimal flap and true and false lumens in the ascending aorta.

**Figure 2:**
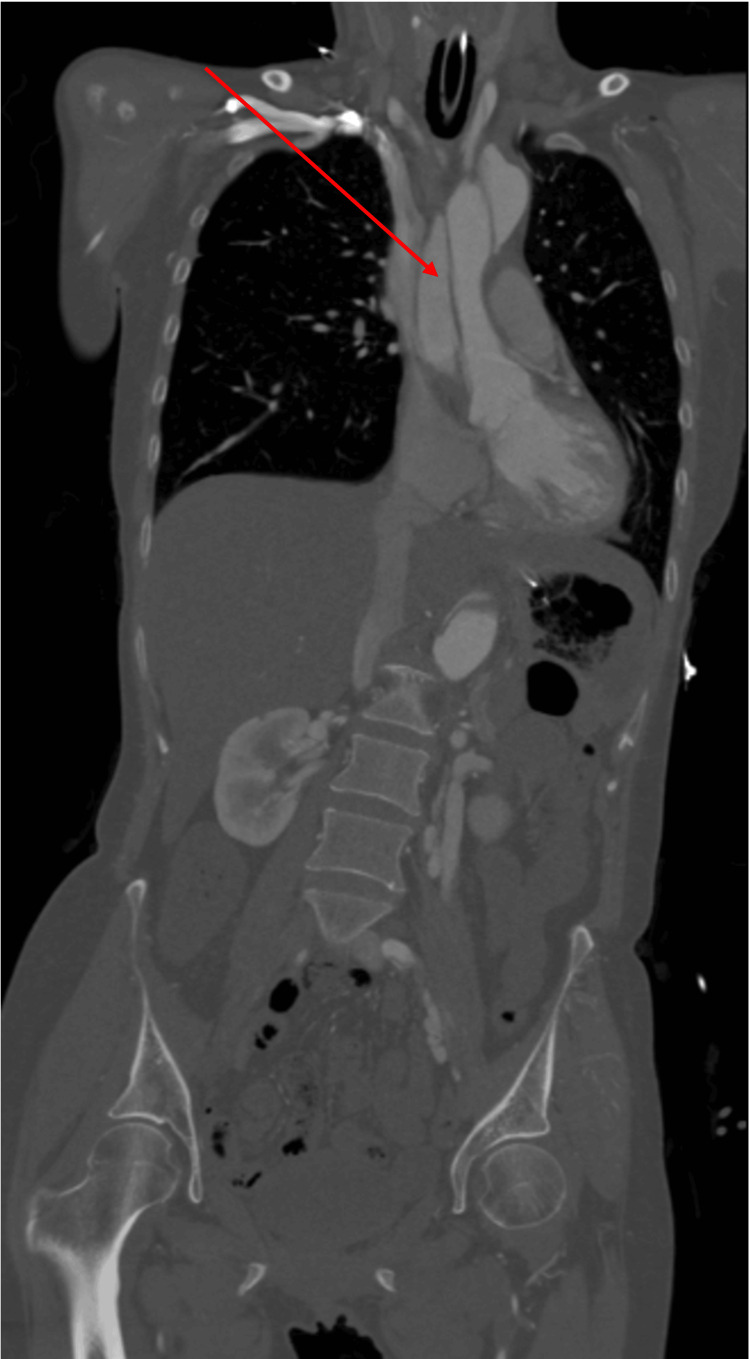
Coronal CT angiogram showing extensive propagation of the dissection from the aortic root through the thoracoabdominal aorta with involvement of major branch vessels.

CT angiography of the chest, abdomen, and pelvis revealed an extensive Stanford Type A aortic dissection extending from the ascending aorta through the descending thoracic aorta and into the iliac arteries. Nicardipine infusion was initiated to maintain systolic blood pressure below 120 mmHg. Cardiothoracic surgery evaluated the patient at the bedside and initially advised conservative management due to extensive cerebral vessel involvement. Following neurologic improvement in the ICU, the same team proceeded with emergent operative repair (Figures 1, 2).

Preoperative labs demonstrated rising liver enzymes (AST 27 → 1117 U/L (reference range: 8 to 33 U/L), ALT 17 → 476 U/L (reference range: 7 to 45 U/L)) and creatinine increasing from 0.82 to 1.04 mg/dL (reference range: 0.5 to 1.1 mg/dL), consistent with evolving hepatic and renal ischemia.

Cardiothoracic surgery proceeded with emergent operative intervention for repair of the ascending aorta. The patient developed massive intraoperative hemorrhage early in the procedure, requiring activation of a massive transfusion protocol with administration of packed red blood cells, fresh frozen plasma, platelets, and cryoprecipitate. Despite aggressive resuscitation, she experienced cardiac arrest refractory to advanced measures and expired intraoperatively.

## Discussion

Ketamine’s sympathomimetic effects result from central catecholamine release, leading to transient hypertension and tachycardia [3]. In individuals with unrecognised aortic wall fragility, this surge in hemodynamic stress may precipitate dissection. While cocaine and amphetamine associated dissections are well documented [1,2], reports implicating ketamine in acute cardiovascular pathology remain exceedingly rare [6]. Prior reports have described cardiovascular toxicity and acute systolic heart failure associated with ketamine exposure, though aortic involvement has not been previously reported [6-8].

Urine toxicology screening was obtained after intubation and initiation of post-intubation analgesia and sedation with fentanyl and midazolam, using a standard hospital immunoassay panel for common drugs of abuse. This panel does not directly test for ketamine, and because ketamine is structurally different from phencyclidine (PCP), it generally does not produce a false-positive PCP result, particularly if concentrations are below assay detection thresholds. Consequently, a negative result does not exclude recent ketamine exposure. Recreational ketamine products may also be adulterated or substituted with novel psychoactive substances or ketamine derivatives, which are not detected by conventional immunoassays. Confirmatory testing with gas or liquid chromatography-mass spectrometry was not performed, representing an additional limitation.

Although causality cannot be definitively established, the presentation occurred in close proximity to suspected ketamine use, with no other known predisposing conditions identified. This case has several limitations, including the absence of confirmatory toxicology beyond standard screening, limited preexisting medical history, and incomplete information regarding chronic substance use. Another limitation involves the absence of reliable collateral information, as the husband only briefly presented to the emergency department and reported the events immediately preceding her collapse. Despite these uncertainties, the case highlights the importance of maintaining diagnostic vigilance in patients presenting with altered mental status, polysubstance exposure, and unexplained hemodynamic instability.

## Conclusions

This case underscores the need for heightened awareness of potential cardiovascular complications related to recreational ketamine use. Ketamine use has been increasing both recreationally and through the growth of outpatient infusion centres for mood and pain disorders. Although major cardiovascular complications from ketamine are uncommon, there have been occasional reports of significant cardiac events following both therapeutic use and recreational exposure. These observations suggest that serious adverse effects, while rare, are possible in a variety of prescribed and recreational ​​​​​​settings.

 While a direct causal relationship cannot be confirmed, the observed temporal association and absence of other clear precipitants warrant further investigation. Additional research is needed to better define ketamine’s cardiovascular risks and potential to precipitate aortic pathology. Emergency physicians should consider aortic dissection in the differential diagnosis of patients with atypical presentations of altered mental status and suspected stimulant or dissociative substance exposure.
